# Hyaluronic acid affects the *in vitro* induction effects of Synthetic PAMPS and PDMAAm hydrogels on chondrogenic differentiation of ATDC5 cells, depending on the level of concentration

**DOI:** 10.1186/1471-2474-14-56

**Published:** 2013-02-05

**Authors:** Katsuhisa Yoshikawa, Nobuto Kitamura, Takayuki Kurokawa, Jian Ping Gong, Yutaka Nohara, Kazunori Yasuda

**Affiliations:** 1 Department of Sports Medicine and Joint Surgery, Graduate School of Medicine, Hokkaido University, Kita-15 Nishi-7, Sapporo, 060-8638, Japan; 2 Department of Orthopedic Surgery, Dokkyo Medical University, 880 Nita-Kobayashi, Mibu-chou, Tochigi-ken 321-0293, Japan; 3 Laboratory of Soft and Wet Matter, Department of Advanced Transdisciplinary Sciences, Faculty of Advanced Life Science, Hokkaido University, Kita-13 Nishi-8, Sapporo 060-0810, Japan

## Abstract

**Background:**

It has been a common belief that articular cartilage tissue cannot regenerate *in vivo*. Recently, however, we have found that spontaneous hyaline cartilage regeneration can be induced *in vivo* by implanting a synthetic double-network (DN) hydrogel, which is composed of poly-(2-acrylamido-2-methylpropanesulfonic acid) (PAMPS) and poly-(N,N’-dimethyl acrylamide) (PDMAAm). However, the mechanism of this phenomenon has not been clarified. Recently, we have found that single-network PAMPS and PDMAAm gels can induce chondrogenic differentiation of ATDC5 cells in vitro even in a maintenance medium. In the *in vivo* condition, there is a strong possibility that the induction effect of the gel itself is enhanced by some molecules which exist in the joint. We have noticed that the joint fluid naturally contains hyaluronic acid (HA). The purpose of this study is to clarify *in vitro* effects of supplementation of HA on the differentiation effect of the PAMPS and PDMAAm gels.

**Methods:**

We cultured the ATDC5 cells on the PAMPS gel, the PDMAAm gel, and the polystyrene (PS) dish surface with the maintenance medium without insulin for 7 days. HA having a molecular weight of approximately 800 kDa was supplemented into the medium so that the concentration became 0.00, 0.01, 0.10, or 1.00 mg/mL. We evaluated the cultured cells with phase-contrast microscopy and PCR analyses.

**Results:**

On the PAMPS gel, supplementation with HA of 0.01 and 0.10 mg/mL significantly increased expression of type-2 collagen mRNA (p = 0.0008 and p = 0.0413) and aggrecan mRNA (p = 0.0073 and p = 0.0196) than that without HA. On the PDMAAm gel, supplementation with HA of 1.00 mg/mL significantly reduced expression of these genes in comparison with the culture without HA (p = 0.0426 and p = 0.0218).

**Conclusions:**

The *in vitro* induction effects of the PAMPS and PDMAAm gels on chondrogenic differentiation of ATDC5 cells are significantly affected by HA, depending on the level of concentration. These results suggested that there is a high possibility that HA plays an important role in the *in vivo* spontaneous hyaline cartilage regeneration phenomenon induced by the PAMPS/PDMAAm DN gel.

## Background

Articular (hyaline) cartilage injury is a significant and increasing health care concern. It has been a common belief that hyaline cartilage tissue cannot regenerate *in vivo *[1,2]. Recently, however, we have found that spontaneous hyaline cartilage regeneration can be induced *in vivo* within a large osteochondral defect in the rabbit at 4 weeks by implanting a synthetic double-network (DN) hydrogel, which is composed of poly-(2-acrylamido-2-methylpropanesulfonic acid) (PAMPS) and poly-(N,N’-dimethyl acrylamide) (PDMAAm) [3]: Namely, we surgically implanted a DN gel plug at the bottom of the defect so that a 1.5 to 3.5-mm deep vacant space was intentionally left within the defect [4,5]. Then, the defect was immediately filled with a blood clot. In the blood clot, a cartilage-like tissue rich in proteoglycan appeared at 2 weeks. At 4 weeks, the defect was filled with the hyaline cartilage tissue [4,5]. Based on these studies, they have proposed a novel therapeutic strategy, *in vivo* spontaneous cartilage regeneration with the DN gel, to treat an articular cartilage defect [6,7]. To clarify mechanisms of the cartilage regeneration phenomenon induced by the PAMPS/PDMAAm DN gel, we have studied the *in vitro* behavior of the chondrogenic ATDC5 cells on single-network PAMPS and PDMAAm gels, which were components of the DN gel, and reported that these gels induce chondrogenic differentiation of ATDC5 cells *in vitro* even in a maintenance medium without the inclusion of insulin [8]. It is remarkable that these synthetic gels have the effect that induces chondrogenic cells to differentiate to chondrocytes, which is similar to insulin. In the *in vivo* condition surrounding the spontaneous hyaline cartilage regeneration induced by the DN gel, however, there is a strong possibility that the induction effect of the gel itself is enhanced by some molecules which exist in the joint. It is important to clarify the effects of such molecules on the induction effect of the PAMPS and PDMAAm gels on chondrogenic differentiation of ATDC5 cells in order to elucidate the mechanisms of the spontaneous cartilage regeneration induced *in vivo* by the DN gel. We have noticed that the joint fluid naturally contains hyaluronic acid (HA), because recent studies have reported that HA improves chondrocyte proliferation and increases matrix synthesis [9-11]. Therefore, we have hypothesized that the *in vitro* induction effects of the PAMPS and PDMAAm gels on chondrogenic differentiation of ATDC5 cells may be significantly affected by HA, depending on the level of concentration. The purpose of this study is to test this hypothesis.

## Methods

### Materials

AMPS (Tokyo Kasei Kogyo), DMAAm (Junsei Chemicals) and 2-oxoglutaric acid (Wako Pure Chemicals, Japan) were purchased and used as is in this study. N,N’-methylenebisacrylamide (MBAA; Tokyo Kasei Kogyo) was purified by recrystallization from ethanol. PAMPS gel and PDMAAm gel were synthesized by radical polymerization. An aqueous solution of 1 mol/L monomer, 4 mol% for PAMPS and 2 mol% for PDMAAm crosslinker, and 0.1 mol% 2-oxoglutaric acid as an initiator were prepared in a reaction cell that had been purged with nitrogen gas for 30 minutes and was irradiated with UV light for 6 hours. All gel reactions were carried out between a pair of glass substrates. After gelation, gel plates were immersed in 4-(−2-hydroxyethyl)-piperazine-1-ethansulfonic acid (HEPES; Sigma) buffer solution (NaHCO3 1.55 × 10-2 M, HEPES 5 × 10–3 M, NaCl 0.14 M, pH 7.4) and the HEPES solution was changed twice daily for 1 week to reach equilibrium. A created gel plate was 2–3 mm thick. Gel disks were made by coring the gel plate with a custum-made hollow punch, which had a cylindrical thin sharp blade having a diameter of 15 mm. Then, the gel discs immersed in the solution were sterilized by autoclaving (120 degrees Celsius, 20 min). In our previous observation, we observed that creation of the disc with the hollow punch and sterilization by autoclaving did not change the surface of each gel [8]. The gel disk was then placed in 24-well polystyrene (PS) dishes for tissue culture.

### Study design

This study consisted of 3 sub-studies: The first sub-study was performed to prove whether the custom-made PAMPS and PDMAAm gels used in the present study could surely induce the chondrogenic differentiation of ATDC5 cells at day 7 in culture with the maintenance medium. The ATDC5 cells cultured on the PAMPS and PDMAAm gels in the maintenance medium were compared with the cells cultured on the polystyrene (PS) dish in the maintenance medium (the negative control) and in the differentiation medium (positive control). The maintenance medium consisted of a 1:1 mixture of Dulbecco’s modified Eagle’s medium and Ham’s F-12 medium (GIBCO) supplemented with 5% fetal bovine serum, 10 mg/ml human transferrin (Roche Molecular Biochemicals), and 3 × 10–8 M sodium selenite (Sigma–Aldrich, St. Louis, MO, USA). The differentiation medium used to induce chondrocyte differentiation of the ATDC5 cells was made by supplementing the maintenance medium with 10 μg/ml of bovine insulin (Sigma–Aldrich). The ATDC5 cell line was obtained from the RIKEN cell bank (Tsukuba, Japan). ATDC5 cells were seeded at a cell density of 5 × 10^4^ cells/cm^2^, and cultured on the PAMPS gel, the PDMAAm gel, and the polystyrene (PS) dish surface at 37°C under 5% CO2 for 7 days. The PS dish was used as the control data, because it was known that the ATDC5 cells cultured on the PS dish in the maintenance medium do not differentiate into chondrocytes.

The second sub-study was performed to clarify whether supplementation of the HA into the above-described maintenance medium significantly affected the *in vitro* induction effects of the PAMPS gel on chondrogenic differentiation of ATDC5 cells. HA having a molecular weight of approximately 800 kDa (ARTZ^®^, Seikagaku Co., Tokyo, Japan) was used in this study. The HA was manufactured in a sterilized condition for medical use (injection into joints) under regulation of Ministry of Health, Labour and Welfare, Japan, and infused into an ampule of 25 mL. For this experiment, we purchased the HA ampule, and supplemented it into the medium so that the concentration became 0.00, 0.01, 0.10, or 1.00 mg/mL. We cultured the ATDC5 cells on the PAMPS gel surfaces in these 4 types of maintenance mediums with or without HA for 7 days.

The third sub-study was performed to clarify whether supplementation of the HA into the maintenance medium significantly affected the *in vitro* induction effects of the PDMAAm gel on chondrogenic differentiation of ATDC5 cells. Using the same maintenance medium containing the HA of 4 concentrations (0.00, 0.01, 0.10, or 1.00 mg/mL), we cultured the ATDC5 cells on the PDMAAm gel surfaces for 7 days.

The time of observation (day 7) was determined according to the results in our foregoing study [8]. Namely, first, ATDC5 cells differentiated into chondrocytes within approximately 7 days, when they were cultured on these gels in the maintenance medium. Secondly, chondrocyte nodules cultured on the PDMAAm gel became larger, and detached spontaneously from the surface after day 10. Therefore, day 7 was the best time to compare the effects of HA supplementation on the cells.

### Evaluation methods

At 7 days of culture, we observed the cultured cells with phase-contrast microscopy, and performed real-time polymerase chain reaction (PCR) analyses for gene expression of type-2 collagen and aggrecan in the cultured ATDC5 cells. The PAMPS and PDMAAm hydrogels were transparent so that it was possible to observe the cells cultured on top of the gel discs using phase contrast microscopy. As for PCR analyses, total RNA was isolated from the ATDC5 cells using the RNeasy mini kit (Qiagen Inc., Valencia, CA). RNA quality from each sample was assured by the A260/280 absorbance ratio. The RNA (100 ng) was reverse-transcribed into single strand cDNA using PrimeScript^®^ RT reagent kit (TakaraBio, Ohtsu, Japan). The sequences of primers used in real time PCR analyses were as follows: type-2 collagen forward AGGGCAACAGCAGGTTCACATAC; reverse TGTCCA CACCAAATTCCTGTTCA. Aggrecan forward AGTGGA TCGGTCTGAATGACAGG; reverse AGAAGTTGTCAG GCTGGTTTGGA. GAPDH forward TGTGTCCGTCGT GGATCTGA; reverse TTGCTGTTGAAGTCGCAGGAG. The real time PCR was performed in Thermal Cycler Dice^®^ TP800 (TakaraBio, Ohtsu, Japan) by using SYBR^®^ Premix Ex TaqTM (TakaraBio, Ohtsu, Japan). cDNA template (5 ng) was used for real time PCR in a final volume of 25 microlitter. cDNA was amplified according to the following condition: 95 degrees Celsius for 5 sec and 60 degrees Celsius for 30 sec at 40 amplification cycles. Fluorescene changes were monitored with SYBR Green after every cycle. A dissociation curve analysis was performed (0.5 degrees Celsius/sec increase from 60 to 95 degrees Celsius with continuous fluorescene readings) at the end of cycles to ensure that single PCR products were obtained. The results were evaluated using the Thermal Cycler Dice^®^ Real Time System software program (TakaraBio, Ohtsu, Japan). The *delta-delta-Ct (ddCt)* algorithm was used to analyze the relative changes in gene expression [12]. Briefly, the data were analyzed using the equation: When time 0 is the first expression of the target gene normalized to that of GAPDH, and time *x* is any time point;

$$\begin{array}{c} \mathrm{ddCt}={\left({\mathrm{Ct}}_{\text{Target}}-{\mathrm{Ct}}_{\mathrm{GAPDH}}\right)}_{\text{Time}\;x}\\ \;-{\left({\mathrm{Ct}}_{\text{Target}}-{\mathrm{Ct}}_{\mathrm{GAPDH}}\right)}_{\text{Time}\;0} \end{array}$$

$$\left[\text{amount}\;\text{of}\;\text{target}\;\text{gene}\right]{=2}^{-\mathrm{ddCt}}$$

### Statistical analysis

In each sub-study, the sample number was six for each analysis: Namely, we calculated the mean and the standard deviation from 6 samples in each group, and compared all the groups in each analysis. The ANOVA was used with the Fisher’s PLSD test for multiple comparisons. The significance limits were set at p = 0.05.

## Results

### Effects of the PAMPS and PDMAAm gel surfaces on ATDC5 cells cultured in the maintenance medium

**Figure 1:**
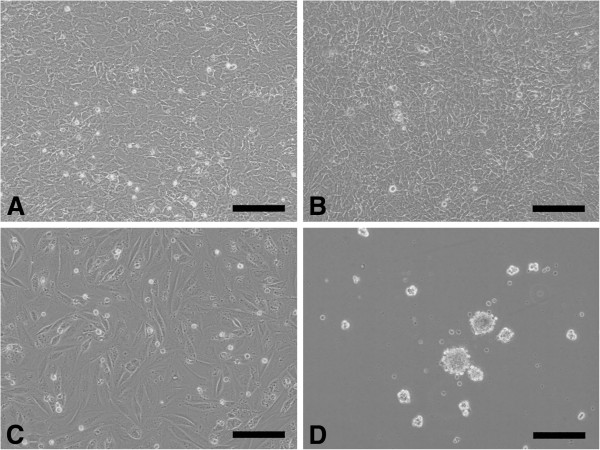
**Histological observations of the ATDC5 cells cultured for 7 days, using phase contrast microscopy (each scale bar shows 100 micrometer). A**: Culture on polystyrene (PS) in the maintenance medium (without insulin); **B**: Culture on PS in the differentiation medium (with insulin); **C**: Culture on PAMPS gel in the maintenance medium; **D**: Culture on PDMAAm gel in the maintenance medium.

In culture on the polystyrene (PS) surface in the maintenance medium without insulin, phase contrast microscopy showed that the cells proliferated to form a confluent monolayer at 7 days (Figure 1A). In cultures on both the polystyrene (PS) surface in the differentiation medium with insulin (Figure 1B) and the PAMPS gel surface in the maintenance medium without insulin (Figure 1C), the cells adhered to the entire surface, and became confluent, although cell aggregation was rarely found at 7 days. The cultured cells attached onto the PDMAAm gel formed nodules (Figure 1D), although many cells did not attach onto the gel surface.

**Figure 2:**
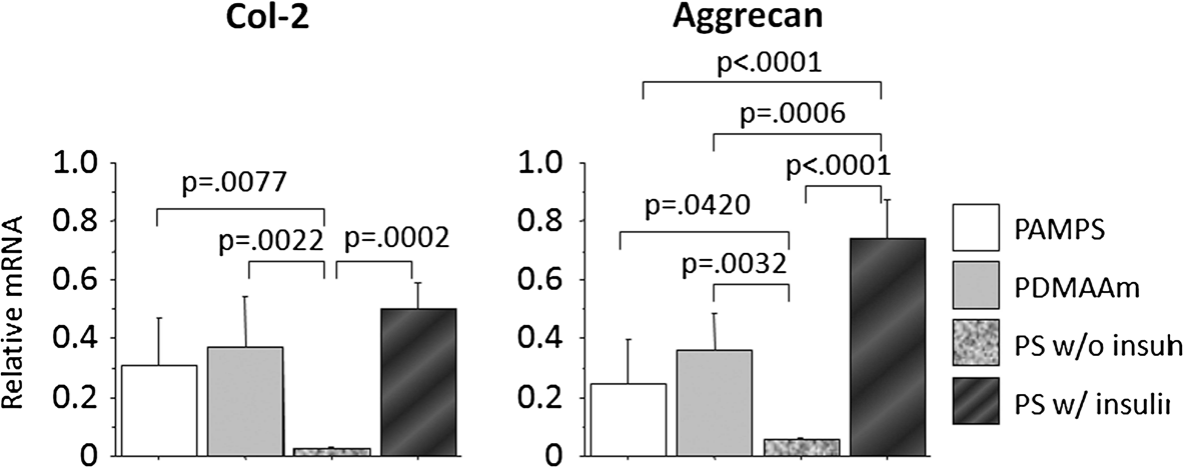
**PCR analyses concerning the effects of the PAMPS and PDMAAm gel surfaces on ATDC5 cells cultured in the maintenance medium.** Each bar shows the mean and the standard deviation from 6 samples. Expression of type-2 collagen (Col-2) and aggrecan genes was significantly greater on the PAMPS and PDMAAm gels than on the polystyrene surface (“PS w/o insulin” shown in the graph), and the former expression levels were comparable to the levels of the cells cultured on the polystyrene surface in the differentiation medium with insulin (“PS w/ insulin” shown in the graph).

PCR analyses showed that expression of type-2 collagen and aggrecan genes was significantly greater on the PAMPS and PDMAAm gels than on the polystyrene (PS) surface (p < 0.05: Figure 2). On the other hand, expression of these genes on the polystyrene surface in the differentiation medium with insulin was significantly greater than that in the maintenance medium (p < 0.05: Figure 2). There was no significant difference in the expression of type-2 collagen gene between the cells cultured on the PAMPS and PDMAAm gels in the maintenance medium and the cells cultured on the polystyrene (PS) surface in the differentiation medium, while expression of aggrecan gene on the polystyrene surface in the differentiation medium with insulin was significantly greater than those on the PAMPS and PDMAAm gels in the maintenance medium (p < 0.0001, p = 0.0006, respectively).

### Influences of hyaluronic acid to the induction effect of PAMPS gel on chondrogenic differentiation of ATDC5 cells

**Figure 3:**
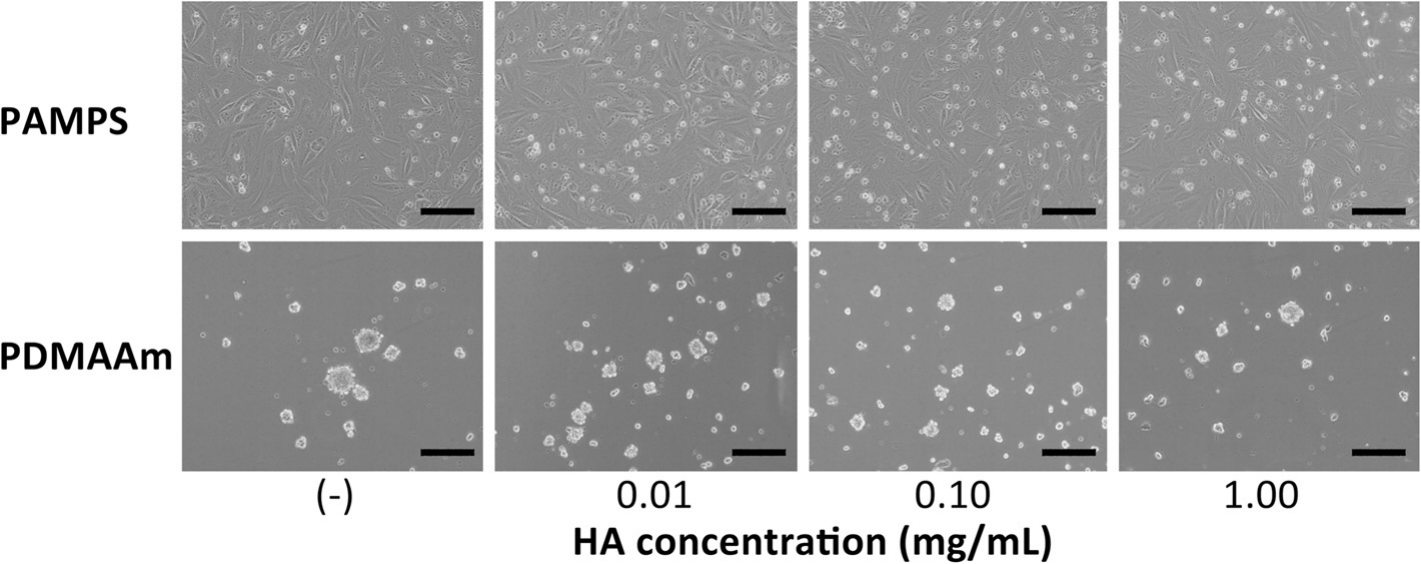
**Histological observations of the ATDC5 cells cultured for 7 days using phase contrast microscopy (each scale bar shows 100 micrometer).** The cells were cultured on PAMPS gel or PDMAAm gel in the maintenance medium without hyaluronic acid (HA) (“-“ shown in the photograph), and in the mediums containing HA of 0.01, 0.10, and 1.00 mg/mL concentrations. On the PAMPS gel, phase contrast microscopy did not show any nodules in each medium. On the PDMAAm gel, we found a tendency that the size of nodules was smaller in the mediums containing HA than in the medium without HA, and that the number of the nodules was less in the mediums containing HA of 1.00 mg/mL concentration than in the other medium with HA.

**Figure 4:**
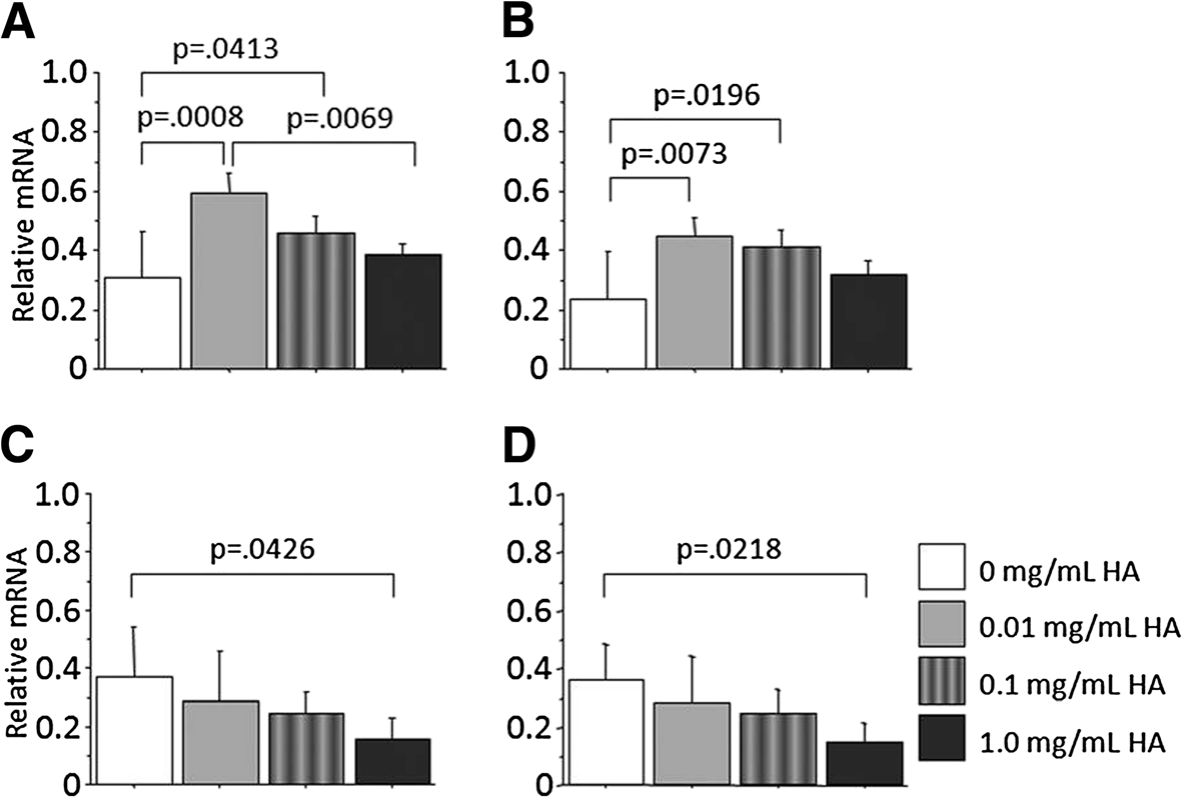
**PCR analyses concerning influences of hyaluronic acid (HA) to the induction effect of PAMPS and PDMAAm gels on chondrogenic differentiation of ATDC5 cells.** Each bar shows the mean and the standard deviation from 6 samples. Concerning the PAMPS gel, supplementation with HA of 0.01 and 0.10 mg/mL significantly increased expression of type-2 collagen gene (**A**) and aggrecan gene (**B**) than that without HA (“0 mg/mL“ shown in the photograph), while supplementation with HA of 1.00 mg/mL did not significantly affect expression of these genes. Regarding PDMAAm, supplementation with HA of 0.01 and 0.10 mg/mL did not significantly affect expression of type-2 collagen (**C**) and aggrecan genes (**D**), while supplementation with HA of 1.00 mg/mL significantly reduced expression of these genes in comparison with the culture without HA.

In culture on the PAMPS gel in the maintenance medium without insulin, phase contrast microscopy did not show any nodules in each medium (Figure 3). However, the PCR analyses demonstrated that supplementation with HA of 0.01 and 0.10 mg/mL significantly increased expression of type-2 collagen mRNA (p = 0.0008 and p = 0.0413) and aggrecan mRNA (p = 0.0073 and p = 0.0196) than that without HA (Figure 4), while supplementation with HA of 1.00 mg/mL did not significantly affect expression of these genes.

### Influences of hyaluronic acid to the induction effect of PDMAAm gel on chondrogenic differentiation of ATDC5 cells

In culture on the PDMAAm gel in the maintenance medium, phase contrast microscopy showed a tendency that the size of nodules was smaller in the mediums containing HA than in the medium without HA, and that the number of the nodules was less in the mediums containing HA of 1.00 mg/mL concentration than in the other medium with HA (Figure 3). The PCR analyses demonstrated that supplementation with HA of 0.01 and 0.10 mg/mL did not significantly affect expression of type-2 collagen mRNA and aggrecan mRNA, while supplementation with HA of 1.00 mg/mL significantly reduced expression of these genes in comparison with the culture without HA (p = 0.0426 and p = 0.0218: Figure 4).

## Discussion

In the first sub-study, we confirmed the basic functions of the custom-made PAMPS and PDMAAm gels used in the present study. Even in the maintenance medium, expression of type-2 collagen and aggrecan genes in the cells cultured on the PAMPS and PDMAAm gels was significantly greater than that in the cells cultured on the polystyrene surface. In addition, the former expression level by the synthetic gels was comparable to the expression level of the cells cultured on the polystyrene surface in the differentiation medium with insulin. These results provided a basis concerning the material function to the second and third sub-studies in the present study. It is remarkable that the synthetic gels can induce chondrogenic differentiation *in vitro* without the existence of any cytokines. No biomaterials having such effects are reported except for these gels. We can speculate that some molecular signals from each hydrogel surface may be delivered to a nucleus of the ATDC5 cells via certain receptors on the cell surface, resulting in induction of chondrogenic differentiation of the cells. In the present study, the effects of the two synthetic gels on the ATDC5 cells were not identical. Therefore, the PAMPS and PDMAAm gels may deliver molecular signals to different receptors on the ATDC5 cell. However, it is unknown what signal transduction system exists between the synthetic gels and the ATDC5 cell. Further molecular biological studies are needed to clarify the signal transduction.

Benya et al. [13] reported that dedifferentiated chondrocytes re-express the differentiated collagen phenotype when cultured on a hydrophobic surface. Namely, they observed that decreased cell attachment promoted collagen type II gene expression. This phenomenon is similar to the behavior of the ATDC5 cells cultured on the PDMAAm gel in the present study. However, the PDMAAm hydrogel is not hydrophobic, because the contact angle to water is 47° [14], while the contact angle a cell culture-treated PS used in the present study is 67°. When the contact angle to water is less than 90°, the surface is hydrophilic. Nevertheless, Yang et al. [14] showed that the dedifferentiated chondrocytes re-express the differentiated collagen phenotype when cultured on the PDMAAm gel. They also showed that the chondrocytes shows low adhesion to the PDMAAm gel, because it is a neutral hydrogel without charged moieties on its polymer chains [14]. We speculate that the similarity in the results between the present study and the Benya’s study may be explained by the low adhesion property of the PDMAAm gel to the chondrogenic cells. On the other hand, the contact angle of the PAMPS gel is only 12 degrees [14], and the PAMPS gel showed high adhesion property to the ATDC5 cells in the present study. Nevertheless, the PAMPS gel showed high ability that differentiated the ATDC5 cells to chondrocytes. This ability cannot be explained by the low adhesion property. It is considered that the mechanism that the PAMPS gel differentiated the ATDC5 cells to chondrocytes is different from the mechanism that the PDMAAm gel did it. Further studies are needed to clarify the mechanism of the PAMPS and PDMAAm gels that can induced chondrogenic differentiation of the ATDC5 cells.

In the second and third sub-studies, we found that the in vitro induction effects of the PAMPS and PDMAAm gels on chondrogenic differentiation of ATDC5 cells were significantly affected by HA, depending on the level of concentration. Namely, supplementation of a relatively low concentration (0.01 and 0.10 mg/mL) of HA significantly enhances the chondrogenic differentiation phenomenon induced by the PAMPS gel. On the other hand, supplementation of a relatively high concentration (1.00 mg/mL) of HA significantly reduced the chondrogenic differentiation phenomenon by the PDMAAm gel. It is known that isolated application of HA to the ATDC5 cells cultured on a polystyrene dish does not induce chondrogenic differentiation. Therefore, these sub-studies suggested that HA supplementation significantly affected the cells which had received certain signals concerning chondrogenic differentiation from the PAMPS or PDMAAm gel. The *in vitro* effects of HA supplementation on the chondrogenic differentiation phenomenon, which was shown in the first sub-study, were different between the PAMPS and PDMAAm gel surfaces. It is known that HA affects chondrocytes through signal transduction receptors that exist on the cell surface, depending on the concentration [15,16]. Because the PAMPS and PDMAAm gels are considered to deliver molecular signals to different receptors on the ATDC5 cell, as implied in the first sub-study, the receptor difference may explain the difference of the HA supplementation effect between these gels.

HA differs from the other glycosaminoglycans, because it has a high molecular weight and it is capable of forming entangled networks. Concerning the effect of HA on chondrogenesis, previous studies reported that HA significantly affects not only gene expression of type-2 collagen and aggrecan but also proteoglycan synthesis in *in vitro* and *in vivo* conditions, depending on the concentration. For example, Akmal et al. [17] reported that supplementation of a low concentration of HA enhances the glycosaminoglycan synthesis in cultured chondrocytes. On the other hand, Allemann et al. [18] studied the effects of HA soaked into a three-dimensional scaffold on bovine chondrocyte function *in vitro*, and reported that a high concentration of HA inhibits the cellularity and matrix accumulation. In addition, recent studies reported that HA has a regulatory role in the maintenance of ESCs in their undifferentiated state *in vitro*[19,20]. Frean et al. [21] demonstrated that HA enhances proteoglycan synthesis of the cartilage tissue, depending on the concentration: a low concentration of HA has stimulatory effects on matrix production, while a high concentration of HA does not show such stimulatory effects. They concluded that there is an optimal concentration in the effect of HA on the chondrogenesis. These reports appear to support the results obtained in the second and third sub-studies.

HA has not only pharmacological effects but also physiochemical effects, the concentration of HA affects its physiochemical property [22]. Concerning the reduction effect of HA with a high concentration, as shown in the third substudy, we can consider a few physical effects of HA. For example, it is known that HA affects cell-substrate adhesion, cell migration, and cell proliferation through signal transducing receptors such as CD44 [23-27]. Therefore, there is a possibility that HA of a high concentration might physically coat the cells and reduce the sensitivity of these receptors, resulting in a reduction of transduction of important molecules related to cell differentiation. Also HA of a high concentration might change the physical conditions of the PDMAAm gel surface, the stiffness of which is closer to the normal cartilage than that of the PAMPS gel. The physical changes might reduce the capability of the PDMAAm gel to induce chondrogenic differentiation of the ATDC5 cells, because the mechanical properties of a material surface significantly affect culture results [28]. Moreover, recent studies reported that HA has a regulatory role in the maintenance of ESCs in their undifferentiated state *in vitro*[19,20]. The regulatory function of HA might maintain or reduce the chondrogenic differentiation of the ATDC5 cells in the case of induction with the PDMAAm gel.

There are some limitations in this study. First, we cultured the cells for only 7 days. As the period for cell differentiation depends on the type of cells, the long term culture may show a different result. Based on our previous study [8], however, we determined that day 7 was the best time to compare the effects of HA supplementation on the cells, as described in the study design section. Therefore, we believe that the 7th day of observation is appropriate for this study. Secondly, we performed PCR analyses concerning only type-2 collagen and aggrecan in the cultured ATDC5 cells, because the over-expression of these mRNAs were accompanied by over-expression of type-2 collagen and aggrecan molecules in the same culture system used in our foregoing study [8]. However, we cannot refer to the effect of HA supplementation on other important molecules, such as SOX9, type-10 collagen, and so on, from this study. Thirdly, we did not count the number of cells cultured on the different discs. For example, although the cultured cells attached onto the PDMAAm gel formed nodules, many cells did not attach onto the gel surface. The data may be explained by the low adhesion property of the PDMAAm gel surface to the ATDC5 cells. Also there is a possibility that HA is inducing more cell detachment from the substrate. Fourthly, we did not assess other culture conditions that might affect chondrogenic differentiation of ATDC5 cells; eg, biomechanical factors, and other stimulating factors. In this study, however, because we intended to focus on the effect of HA concentration on chondrogenic differentiation, we believe that this study design is acceptable. Fifthly, we did not clarify the effect of HA on other culture systems including different cells. Thus, further studies will be needed to clarify the effect of HA supplementation on chondrogenic differentiation induced not only by the PAMPS and PDMAAm gels but also other synthetic materials in the near future. However, the present study has provided important evidence that *in vitro* HA supplementation can significantly modify the chondrogenic differentiation induced by the PAMPS and PDMAAm gels in a complex manner, depending on the concentration.

## Conclusion

On the PAMPS gel, supplementation with HA of 0.01 and 0.10 mg/mL significantly increased expression of type-2 collagen mRNA (p = 0.0008 and p = 0.0413) and aggrecan mRNA (p = 0.0073 and p = 0.0196) than that without HA. On the PDMAAm gel, supplementation with HA of 1.00 mg/mL significantly reduced expression of these genes in comparison with the culture without HA (p = 0.0426 and p = 0.0218). The present study demonstrated that the *in vitro* induction effects of the PAMPS and PDMAAm gels on chondrogenic differentiation of ATDC5 cells are significantly affected by HA, depending on the level of concentration. These results suggested that there is a high possibility that HA plays an important role in the *in vivo* spontaneous hyaline cartilage regeneration phenomenon induced by the PAMPS/PDMAAm DN gel.

## Competing interests

We have no financial or non-financial competing interests. We do not hold or are not currently applying for any patents relating to the content of the manuscript.

## Authors’ contribution

KY performed the experiment and the microscopic observations. NK performed the RT-PCR analysis. YN contributed to the data analysis. TK and JPG created the DN-gel material. KY designed the study and drafted the manuscript. All authors read and approved the final manuscript.

## Pre-publication history

The pre-publication history for this paper can be accessed here:

http://www.biomedcentral.com/1471-2474/14/56/prepub
